# The Vestibular Implant Input Interacts with Residual Natural Function

**DOI:** 10.3389/fneur.2017.00644

**Published:** 2017-12-14

**Authors:** Raymond van de Berg, Nils Guinand, Maurizio Ranieri, Samuel Cavuscens, T. A. Khoa Nguyen, Jean-Philippe Guyot, Florence Lucieer, Dmitrii Starkov, Herman Kingma, Marc van Hoof, Angelica Perez-Fornos

**Affiliations:** ^1^Division of Balance Disorders, Faculty of Health Medicine and Life Sciences, Department of Otorhinolaryngology and Head and Neck Surgery, School for Mental Health and Neuroscience, Maastricht University Medical Center, Maastricht, Netherlands; ^2^Faculty of Physics, Tomsk State University, Tomsk, Russia; ^3^Service of Otorhinolaryngology and Head and Neck Surgery, Department of Clinical Neurosciences, Geneva University Hospitals, Geneva, Switzerland; ^4^Translational Neural Engineering Lab, Center for Neuroprosthetics, Interfaculty Institute of Bioengineering, École Polytechnique Fédérale de Lausanne, Lausanne, Switzerland

**Keywords:** vestibular implant, vestibular prosthesis, neural prosthesis, bilateral vestibular areflexia, bilateral vestibulopathy, vestibulo-ocular reflex

## Abstract

**Objective:**

Patients with bilateral vestibulopathy (BV) can still have residual “natural” function. This might interact with “artificial” vestibular implant input (VI-input). When fluctuating, it could lead to vertigo attacks. Main objective was to investigate how “artificial” VI-input is integrated with residual “natural” input by the central vestibular system. This, to explore (1) whether misalignment in the response of “artificial” VI-input is sufficiently counteracted by well-aligned residual “natural” input and (2) whether “artificial” VI-input is able to influence and counteract the response to residual “natural” input, to show feasibility of a “vestibular pacemaker.”

**Materials and methods:**

Five vestibular electrodes in four BV patients implanted with a VI were available. This involved electrodes with a predominantly horizontal response and electrodes with a predominantly vertical response. Responses to predominantly horizontal residual “natural” input and predominantly horizontal and vertical “artificial” VI-input were separately measured first. Then, inputs were combined in conditions where both would hypothetically collaborate or counteract. In each condition, subjects were subjected to 60 cycles of sinusoidal stimulation presented at 1 Hz. Gain, asymmetry, phase and angle of eye responses were calculated. Signal averaging was performed.

**Results:**

Combining residual “natural” input and “artificial” VI-input resulted in an interaction in which characteristics of the resulting eye movement responses could significantly differ from those observed when responses were measured for each input separately (*p* < 0.0013). In the total eye response, inputs with a stronger vector magnitude seemed to have stronger weights than inputs with a lower vector magnitude, in a non-linear combination. Misalignment in the response of “artificial” VI-input was not sufficiently counteracted by well-aligned residual “natural” input. “Artificial” VI-input was able to significantly influence and counteract the response to residual “natural” input.

**Conclusion:**

In the acute phase of VI-activation, residual “natural” input and “artificial” VI-input interact to generate eye movement responses in a non-linear fashion. This implies that different stimulation paradigms and more complex signal processing strategies will be required unless the brain is able to optimally combine both sources of information after adaptation during chronic use. Next to this, these findings could pave the way for using the VI as “vestibular pacemaker.”

## Introduction

Bilateral vestibulopathy (BV) is defined as severely reduced or totally absent function of the bilateral vestibular organs, vestibular nerves or a combination of both (1). Associated symptoms are postural instability, blurred vision (oscillopsia) and impaired spatial orientation abilities (2, 3). Up to 84% of the patients report a significant reduction in quality of life and there are considerable physical and socioeconomic impacts (4, 5). Since central vestibular compensation and sensory substitution are often not sufficient to counterbalance the lack of vestibular information (6), prognosis is poor in most cases (7). Therapeutic options are limited and remain ineffective for high-frequency and unpredictable movements (8, 9). In this context, restoring vestibular function using a vestibular implant (VI) might be beneficial for BV patients. Many research groups around the world are now investigating the feasibility, technical aspects and biomechanical issues of this option (10–13). The first results of a motion-modulated vestibular prosthesis in humans were previously published by the Geneva-Maastricht group and provided clear evidence for the feasibility of a clinically useful VI in humans (14–18).

The prototype implants investigated in animals and humans still have many challenges to overcome. A key challenge is to design, surgically implant and adjust the VI in such a way that the desired electrically evoked eye movements closely mimic the characteristics (e.g., gain, angle, phase) of the “natural” vestibulo-ocular-reflex (VOR) response observed upon motion stimuli (15, 17, 19–24). In order to achieve this, two factors might be relevant. First, VI-stimulation can show significant misalignment in the eye movement response as a result of current spread from the electrode location to adjacent nerves (2, 15, 17). Second, residual “natural” input can still be present in BV (7). This latter includes residual vestibular function and extravestibular cues such as proprioception (25). This residual “natural” function might interact with the “artificial” VI-input, possibly influencing the response to the “artificial” VI-input. A fluctuating residual “natural” function could also give complaints (e.g., attacks of vertigo). It has been hypothesized that the VI could counteract the fluctuating residual “natural” function, and serve as a “vestibular” pacemaker (10). Therefore, a crucial point to be investigated in VI research is how this “natural” input (e.g., residual vestibular function as well as extravestibular cues) interacts with the “artificial” VI-input, to generate vestibular responses (i.e., the combined VOR). From a basic science point of view, this could facilitate basic knowledge about how the central vestibular system integrates information of these two inputs. From a clinical point of view, it could facilitate knowledge about how the central vestibular system copes with misalignment of the “artificial” VI-response in the presence of well-aligned residual “natural” input. This could help determining whether a specific stimulation paradigm is needed to correct for the misalignment. Next to this, it could facilitate knowledge about whether the “artificial” VI-input is able to influence the response to residual “natural” input. This could pave the way for using the VI as a “vestibular pacemaker” in the future (10).

Literature on this matter is still scarce in this relatively novel field. In previous animal investigations, vestibular function was ablated in a broad frequency range by canal plugging or ototoxic medication. In such experimental settings, only little vestibular function was preserved (11, 21, 26–28). Therefore, the interactions between “artificial” VI-input and residual “natural” input have not been completely investigated. In previous human investigations conducted by the Geneva-Maastricht group (14, 17, 29), no interactions between the “artificial” and the “natural” inputs were investigated either. The current study was therefore designed to fill this gap by exploring how the “artificial” VOR generated by the VI, is modulated by the interaction between “artificial” VI-input and “natural” residual input during stimulation trials in the first hours after activating the VI (acute activation). In order to answer the clinical relevant questions mentioned above, predominantly horizontal residual “natural” input was combined with “artificial” VI-input that was congruent (predominantly horizontal) and incongruent (predominantly vertical, and inversed).

## Materials and Methods

### Patients and Device

This study was conducted in four BV patients implanted with a modified cochlear implant incorporating three vestibular electrodes (MED-EL, Innsbruck, Austria). The inclusion criteria, surgical procedures and device characteristics were previously described (14, 15, 17). The vestibular electrodes were located at various anatomical sites: two electrodes were implanted in the vicinity of the lateral ampullary nerve (LAN), two electrodes were implanted in the vicinity of the superior ampullary nerve (SAN), and one was implanted in the vicinity of the posterior ampullary nerve (PAN) (Table 1). These tested electrodes will be referred to as: BV1-LAN, BV1-SAN, BV2-PAN, BV3-LAN, and BV4-SAN. Note that electrodes implanted at different anatomical sites were purposefully used in the experiments in order to be able to study the interaction between predominantly horizontal residual “natural” input with both horizontal (LAN stimulation) and vertical (SAN and PAN stimulation) “artificial” VI-input (see Introduction and Study Design and Experimental Procedure).

**Table 1:** Main characteristics of the tested bilateral vestibulopathy-patients with a modified cochlear implant.

Subject	Sex	Etiology	Age at implantation	Surgical approach	Tested electrode(s) and side of implantation	Baseline and modulation amplitude
BV1	F	Trauma	67	Intralabyrinthine	LAN—left side	250 µA ± 30 µA
					SAN—left side	400 µA ± 75 µA
BV2	F	Meningitis	48	Intralabyrinthine	PAN—right side	150 µA ± 50 µA
BV3	M	DFNA-9	67	Intralabyrinthine	LAN—left side	120 µA ± 60 µA
BV4	M	Trauma	53	Intralabyrinthine	SAN—right side	350 µA ± 125 µA

### Electrical Stimulation

As previously described, baseline stimulation of the vestibular nerve was restored in order to be able to generate bidirectional eye movements (i.e., upwards/downwards when stimulating the vertical nerve branches, and rightwards/leftwards when stimulating the lateral ampullary branch) with only unilateral vestibular stimulation (14, 15, 29). In this study a supraphysiological baseline was used, consisting of constant amplitude trains of biphasic charge-balanced pulses (200 μs/phase) presented at a rate of 400 pulses per second. The amplitude was set in the middle of the dynamic range measured for each patient (15). This has shown to be effective in generating bidirectional vestibular sensations with a unilateral prosthesis (2, 15). It was previously reported that activating an implant does not often result in major discomfort and that nystagmus disappears within minutes, especially after repeated “on-off” transitions (15, 29). Therefore, it was waited until the subjects were in the adapted state (e.g., when the spontaneous nystagmus had disappeared) (29). Then an electrical signal was used to up- and down-modulate the amplitude of the train of pulses delivered by the vestibular electrodes. The modulation signal was generated by a 3D gyroscope (LYPR540AH; ST Micro-electronics; Geneva, Switzerland) fixated to a velocity-controlled rotatory chair (Nystagliner Pro; Erich Jaeger Gmbh) used to deliver precise sinusoidal rotations in the horizontal plane. For short, gyroscopes only capturing yaw-plane motion of the rotatory chair, served as input for modulation. Modulation was then applied to the electrodes located in horizontal as well as vertical semicircular canals. Therefore, yaw-plane motion led to an electrical response in all implanted semicircular canals. In this study, a linear transfer function was used in which the modulation strength (i.e., function slope) was chosen in such a way that at the fastest motion stimuli (30°/s, see experimental procedure below) the amplitude of electrical stimulation corresponded to 50–90% of the dynamic range of that specific electrode. This characteristic remained constant during the experiments. Note that this stimulation paradigm implied symmetric or equal modulation for excitatory and inhibitory stimuli (14, 17). The specific electrical stimulation details for each tested electrode in each patient are presented in Table 1.

### Study Design and Experimental Procedure

Patients were tested in a controlled laboratory setting, in complete darkness. They were instructed to sit still, keep their head straight up (not fixed), look in front of them and keep their eyes open during the trials. As the trials required substantial time, it was chosen to not fixate the patients’ head on the chair or to use biteboard fixed gyroscopes. Therefore, it was first confirmed in a control experiment that, at the relative low rotational amplitudes and frequencies used in this study, head motion closely followed the motion stimulation profile. In addition, alerting tasks (e.g., counting down from 100 by 7, to name boy names starting with an “A,” etc.) were given to all subjects during experimental trials to improve level of concentration and arousal.

During experimental trials, patients were subjected to 60 sinusoidal cycles with a peak velocity of 30°/s and presented at a frequency of 1 Hz, delivered by the velocity-controlled rotatory chair. Trials were performed in four experimental conditions:

1. *VOR condition*: patient sitting on the rotatory chair (i.e., subject to horizontal whole-body rotations), without electrical stimulation. This condition was used to quantify the patients’ residual “natural” vestibular function in horizontal plane (including any contribution of extravestibular cues).
2. *Electrically evoked VOR condition (eVOR condition)*: patient sitting aside in an immobile chair (static, no motion) while the amplitude of the electrical stimulation delivered through one vestibular electrode was modulated by the gyroscope fixed to the rotatory chair. Since electrodes at different anatomical sites were used, the eVOR response could be cross-axial to the residual “natural” response measured in the VOR condition. The eVOR condition, as well as the paradigm of electrical stimulation, has been previously described (17). This condition was designed to quantify the VOR response generated exclusively by VI-input (i.e., no contribution of residual “natural” and/or extravestibular cues).
3. *Total VOR condition with “regular” modulation (totalVOR*+*)*: patient sitting on the rotatory chair (i.e., subject to horizontal whole-body rotations), while the amplitude of the electrical stimulation delivered through one vestibular electrode was modulated by the gyroscope fixed to the rotatory chair [see also (14)]. In this condition, the alignment of the gyroscopes corresponded to the side of implantation. For example, during whole-body rotations to the left, the VI provided an excitatory stimulus for patients implanted on the left and an inhibitory stimulus for patients implanted on the right. Whole-body rotations to the right led to the opposite. This condition was designed to quantify the VOR response when the residual “natural” input and the “artificial” VI-input worked together to generate the response.
4. *Total VOR condition with inversed modulation (totalVOR*−*)*: this experimental condition was similar to the totalVOR+ condition, except that the orientation of the gyroscopes was reversed for the horizontal plane: Instead of delivering an excitatory stimulus during a whole-body rotation to the implanted side, an inhibitory stimulus was delivered by the VI. An excitatory stimulus was elicited by a rotation to the opposite side. In this condition, the “artificial” VI-input could hypothetically counteract the input of the residual “natural” and extravestibular inputs.

The testing sequence was identical for all patients since the after-effects of VI-stimulation are still undetermined (28, 30): VOR, eVOR, totalVOR+, and finally totalVOR−. Trials were repeated if necessary to obtain as many reliable results as possible. Indications for repeating a trial were equal to those used in a clinical setting (3, 31).

### Data Acquisition and Preprocessing

Bidimensional eye movements were recorded during experimental trials using the EyeSeeCam system (EyeSeeTec; Munich, Germany) at a sampling rate of 220 Hz. The eye movement signal was then preprocessed off-line using Matlab R2011b (The Mathworks, Natick, MA, USA). The eye position signal was smoothed first using an 11th order Sawitzky-Golay filter, followed by an 11th order median filter. Then, the signal was differentiated to obtain the eye velocity. Blinks, saccades and quick phases were detected as segments where eye acceleration was above 1,000°/s and eye velocity was above 600°/s, and subsequently removed. Piecewise cubic Hermite interpolation was used to fill the missing values. Finally, eye velocity, head velocity, and time data were then transferred to Mathematica 10.4 (Wolfram Research, Champaign, IL, USA), where any cycles with remaining blink artifacts were manually removed in consensus by two authors (Raymond van de Berg and Dmitrii Starkov).

### Data Analysis of the Remaining Cycles

**Figure 1:**
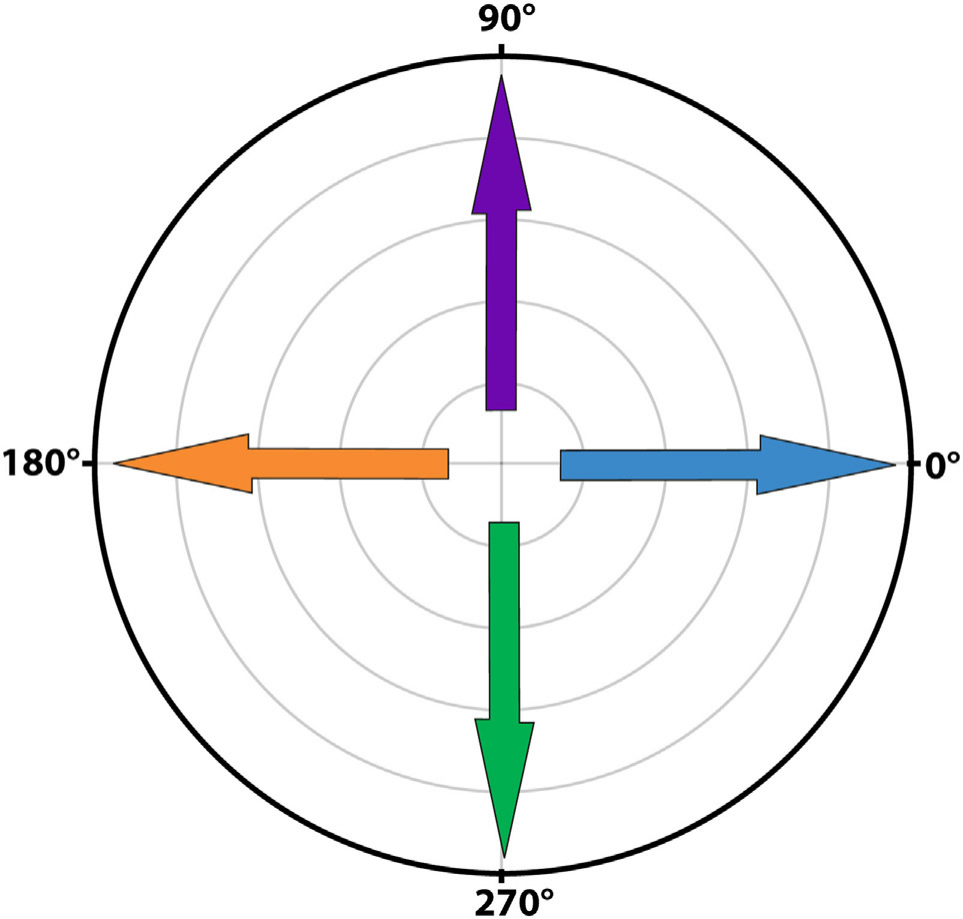
Polar plot illustrating the angles of eye movements. A horizontal eye movement to the right corresponded to an angle of 0^o^ (blue arrow), a horizontal eye movement to the left to an angle of 180^o^ (orange arrow). A completely vertical eye movement upwards corresponded to an angle of 90^o^ (purple arrow) and a completely vertical eye movement downwards to an angle of 270^o^ (green arrow).

Signal averaging was performed by calculating the mean of all cycles for each sample point of the mean cycle. To visualize the average signals, results were plotted separately for horizontal and vertical eye and head velocities. After a first qualitative analysis by three authors (Raymond van de Berg, Marc van Hoof, and Herman Kingma), it was decided to develop a signal analysis algorithm based on peak total eye and peak total head velocities. These were calculated as the square roots of the sums of the squares of horizontal and vertical eye and head velocities. Peak total eye velocities and peak total head velocities per cycle were determined using a peak detection paradigm where the maximum excitatory and inhibitory velocities were identified. After detecting the first peak, the second peak was selected using a weighted distance and amplitude function in relationship to the first peak and to half of the cycle [amplitude × (amount of samples to first peak/amount of samples of half the cycle)]. Cycles were only included if an inhibitory and excitatory peak were present. Gain of each cycle was calculated by dividing peak total eye velocity by peak total head velocity, not corrected for time delay. Since the eVOR condition did not contain sinusoidal head movements (the head was kept stationary) and to allow comparison, the gain in the eVOR condition was based on the peak total eye velocity divided by a hypothetical 1 Hz sinusoidal head movement with a peak velocity of 30^o^/s. The angles for peak total eye and peak total head velocities for each cycle were calculated as the angles between the horizontal and vertical eye and head velocities, in the whole 0–360°circular range (see Figure 1) (17). Asymmetry of each cycle was determined by (excitatory peak total eye velocity − inhibitory peak total eye velocity)/(excitatory peak total eye velocity + inhibitory peak total eye velocity). Phases for each cycle were calculated using a Spearman correlation during cyclic shifting of all samples. The maximums were determined for a shift of half a cycle in both directions (positive and negative). Horizontal and vertical eye and head velocities were used for correlation. Since horizontal eye movements were compared to horizontal and vertical eye movements, horizontal head velocities to the right were considered in phase with horizontal eye velocities to the right and vertical eye velocities upwards. Horizontal head movements to the left were considered in phase with horizontal eye movements to the left and vertical eye movements downwards. Only the predominant phase was plotted (horizontal in BV1-LAN and BV1-SAN, vertical in BV4-SAN), unless horizontal and vertical components were both clearly represented (BV2-PAN and BV3-LAN).

### Statistics

Medians and interquartile ranges were determined. Confidence intervals for medians were bootstrapped 1,000 times. *p*-Values were calculated using the Mann–Whitney *U*-test for the medians. Bonferroni correction was applied (alpha value of 0.05 divided by 36 to correct for multiple testing). An alpha value of 0.0014 was thus considered statistically significant.

### Ethical Considerations

This study was in accordance with the Declaration of Helsinki (amended version 2013). Approval was obtained from the ethical committees of Maastricht University Medical Center (NL36777.068.11/METC 11-2-031) and Geneva University Hospitals (NAC 11-080). All participants provided written informed consent prior to the study.

## Results

### Qualitative Analysis of Signal Shapes

**Figure 2:**
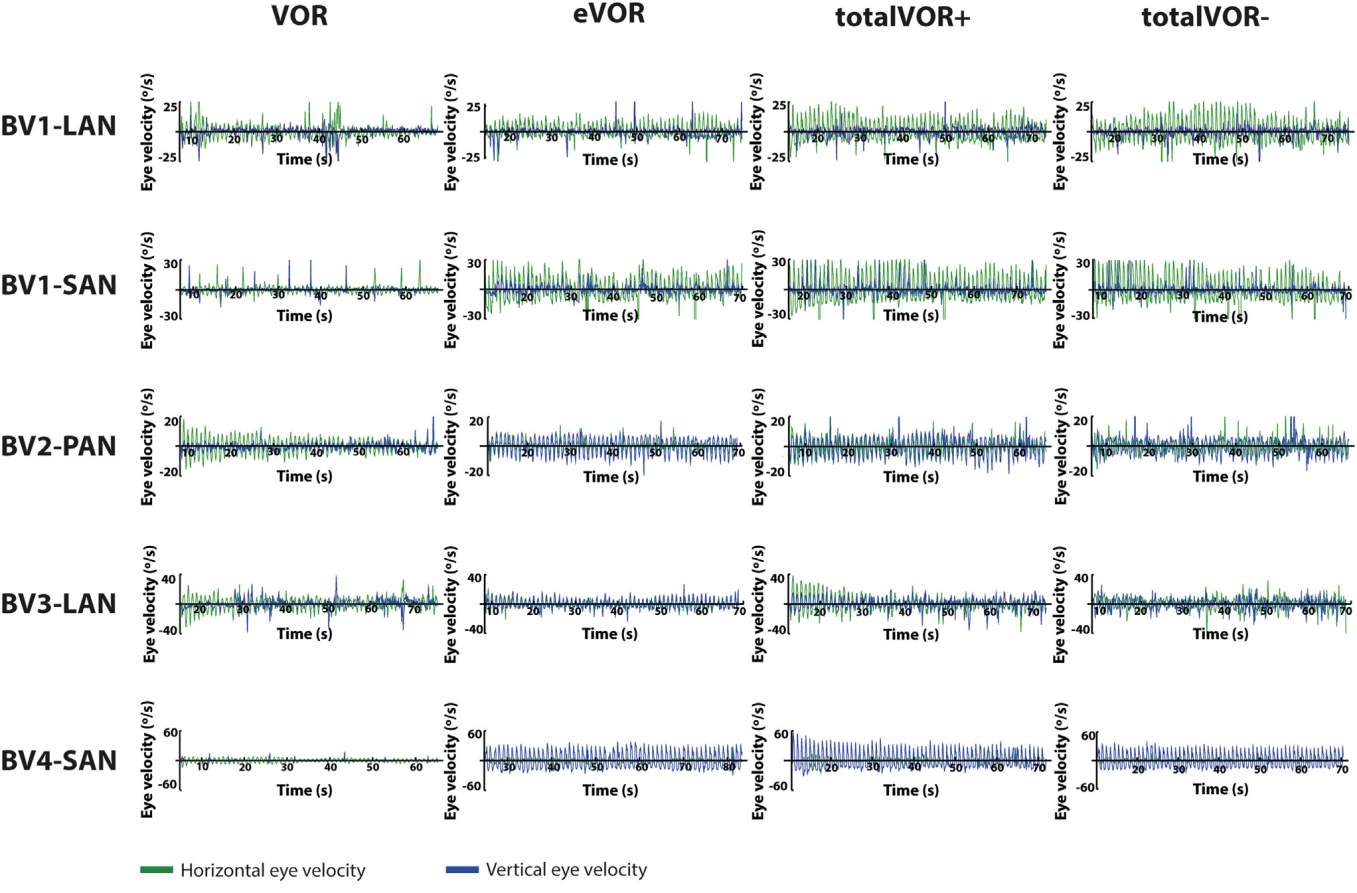
Plots presenting for all subjects the raw eye movement signals of each condition in the horizontal and vertical planes. Positive horizontal velocities correspond to movements to the right and negative horizontal velocities to movements to the left. Positive vertical velocities correspond to movements upwards and negative vertical velocities to movements downwards.

**Figure 3:**
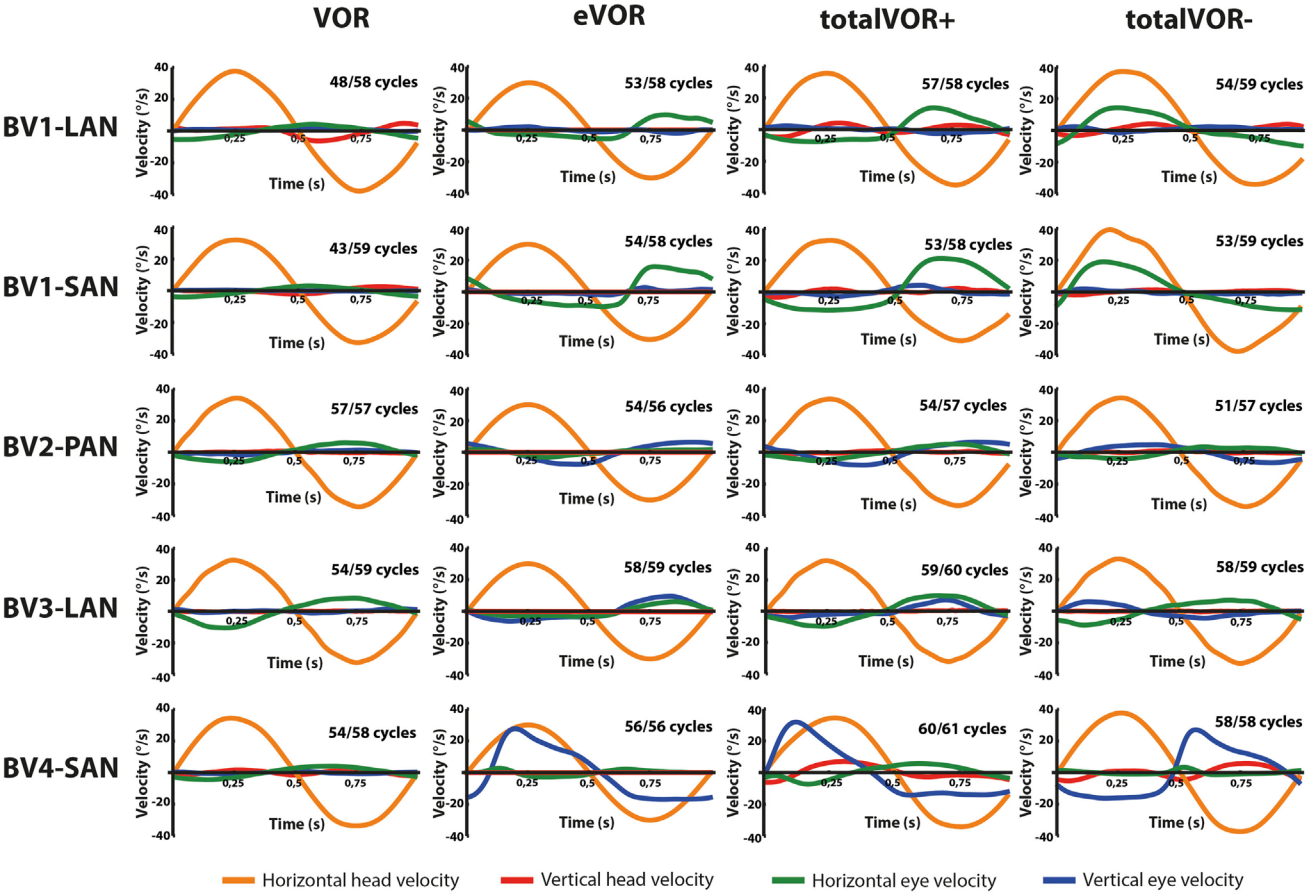
Averaged eye and head movement signals of each condition in the horizontal and vertical planes. Positive horizontal velocities correspond to movements to the right and negative horizontal velocities to movements to the left. Positive vertical velocities correspond to movements upwards and negative vertical velocities to movements downwards. Note that since the electrically evoked vestibulo-ocular-reflex (eVOR) condition involved no head movements, a hypothetical horizontal head movement is plotted corresponding to the electrical stimulus of the vestibular implant. The amount of cycles measured is given, as well as the number of cycles available for analysis after data cleaning.

Figures 2 and 3 present the preprocessed eye and head movement signals of each condition for each subject, before and after averaging. Pure “artificial” VI-input (eVOR condition) could lead to non-linearities in the eye movement response: sinusoidal electrical stimulation did not often evoke sinusoidal eye movements (e.g., eVOR of BV1-LAN, BV1-SAN, BV4-SAN). These non-linearities involved asymmetries between the excitatory phase and the inhibitory phase of stimulation. For example, the eVOR condition for BV4-SAN showed a high peak in the vertical eye velocity during the excitatory phase but a lower and less pronounced peak in the inhibitory phase. Asymmetrical responses were also often observed in the combined totalVOR+ and totalVOR− conditions (e.g., BV1-SAN).

### Characteristics of the Responses Obtained in the Different Experimental Conditions

#### Gain

**Figure 4:**
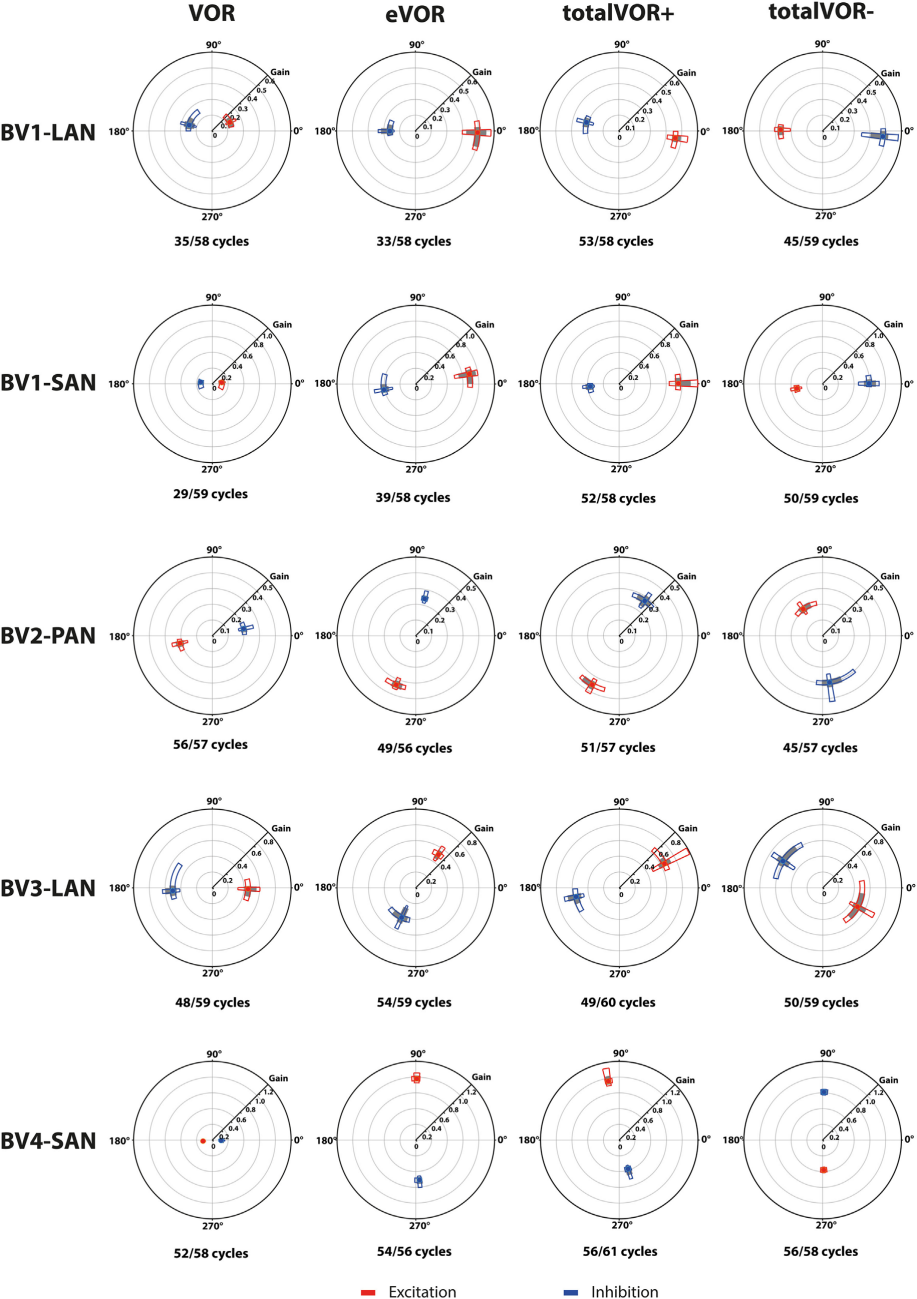
Vectors of peak total eye velocities in the excitatory and inhibitory phases of stimulation, plotted for each electrode in each condition [vestibulo-ocular-reflex (VOR), electrically evoked vestibulo-ocular-reflex (eVOR), totalVOR+, and totalVOR−]. The gain is represented by the vector magnitude. The angle of the response is represented by the vector angle (according to the polar plot in Figure 1). Dots represent the medians, gray bars the 95% confidence intervals and the open bars the interquartile ranges of the vectors of peak total eye velocities. Red represents peak total eye velocities obtained during the excitatory phases of stimulation, blue during the inhibitory phases. The amount of analyzed excitatory and inhibitory peak total eye velocities is given, as well as the amount of peaks available for analysis after data cleaning. Note that, to improve visibility, the scale of the polar plots for each subject was optimized for individual responses and consequently is not uniform across subjects.

Table 2 and Figure 4 present, respectively, the median peak total eye velocities and the vectors of the obtained eye movements in the excitatory and inhibitory phases of stimulation, plotted for each electrode in each condition. As expected, little residual “natural” function (VOR condition) was present in all cases. The median gain value was ≤0.25 for all cases, except for BV3-LAN where it reached around 0.4 for both phases of stimulation.

**Table 2:** Median peak total eye velocities (°/s) and confidence intervals in the excitatory and inhibitory phases of stimulation, of each electrode in each condition.

	Excitation				Inhibition			
	VOR	eVOR	totalVOR+	totalVOR−	VOR	eVOR	totalVOR+	totalVOR−
BV1-LAN	5.7 (5.0–7.2)	14.2 (11.6–16.2)	15.0 (11.6–16.2)	11.3 (10.3–11.0)	6.7 (5.1–8.1)	5.8 (5.4–8.0)	9.4 (8.2–10.3)	16.8 (15.2–18.6)
BV1-SAN	4.3 (3.8–4.9)	20.7 (16.6–23.2)	24.9 (22.8–28.0)	12.4 (11.4–13.3)	4.8 (4.2–5.3)	12.3 (10.4–13.4)	13.1 (11.8–13.9)	23.3 (20.9–27.3)
BV2-PAN	7.2 (6.4–8.3)	10.0 (9.2–10.5)	12.2 (11.6–12.8)	7.1 (6.4–7.7)	6.7 (6.6–7.7)	7.2 (6.8–7.8)	9.2 (8.2–10.0)	10.2 (9.7–11.3)
BV3-LAN	11.7 (10.3–13.3)	12.3 (10.8–13.5)	16.6 (15.1–18.7)	12.9 (11.3–16.5)	13.0 (11.9–16.1)	10.0 (6.8–12.2)	14.5 (12.6–17.2)	16.1 (13.6–18.2)
BV4-SAN	4.6 (4.1–5.4)	28.1 (27.1–29.5)	32.7 (31.1–34.8)	17.3 (16.3–18.1)	4.4 (3.8–5.8)	18.3 (17.5–19.9)	16.3 (15.0–17.4)	28.0 (27.4–28.5)

*VOR, vestibulo-ocular-reflex; eVOR, electrically evoked vestibulo-ocular-reflex*.

When residual “natural” function was combined with “artificial” VI-input, gains increased in totalVOR+ and totalVOR− in all cases, except a non-significant decrease of gain in the excitatory phase of totalVOR− in BV2-PAN. The increases were significant (*p* < 0.00001) for both the excitatory and the inhibitory phases of stimulation of totalVOR+ and totalVOR− in BVL1-LAN, BVL1-SAN, and BV4-SAN. In BV2-PAN, the increase was significant for both phases in totalVOR+, and the inhibitory phase in totalVOR− (*p* ≤ 0.0001). Only the gain in the excitatory phase of stimulation for the totalVOR+ condition was significantly higher (*p* = 0.0004) than VOR for BV3-LAN. When comparing responses of “artificial” VI-input only (eVOR) to the combined conditions totalVOR+ and totalVOR−, gain could increase or decrease with respect to eVOR. These changes were significant in BV1-LAN and BV1-SAN in totalVOR− for both phases (*p* ≤ 0.0002), but not in totalVOR+. In BV2-PAN only the gain of the excitatory phase of totalVOR− was significantly different from eVOR (*p* < 0.00001). In BV3-LAN the gains of the inhibitory phases of totalVOR+ and totalVOR− were significantly different from eVOR (*p* ≤ 0.0002), but not the excitatory phases. In BV4-SAN, both totalVOR+ and totalVOR− were significantly different from eVOR (*p* < 0.00001), except the excitatory phase of totalVOR+. In all electrodes, the excitatory phase always showed a higher gain than the inhibitory phase in totalVOR+. In totalVOR− this was always the opposite. To summarize, gain often significantly changed when combining residual “natural” function with “artificial” VI-input, compared to residual “natural” function only.

#### Angles

Three electrodes elicited eye movement responses that were in the plane of the stimulated canal: a predominantly horizontal response in BV1-LAN, and predominantly vertical responses in BV2-PAN and BV4-SAN. Two other electrodes showed clear misalignment: BV1-SAN elicited a predominantly horizontal response, and BV3-LAN elicited a mixed horizontal and vertical response (Table 3 and Figure 4). When combining residual “natural” input and “artificial” VI-input in totalVOR+, the angles remained predominantly horizontal in BV1-LAN and BV2-SAN. In BV2-PAN, BV3-LAN, and BV4-SAN predominantly horizontal eye movements of VOR were combined with predominantly vertical eye movements of eVOR. This resulted in a shift of the predominantly vertical median peak total eye velocities of eVOR, to the horizontal axis. When inverting the gyroscopes of the VI in totalVOR−, different responses were obtained. In BV1-LAN, BV1-SAN, and BV4-SAN, inversion of the gyroscopes resulted in an almost 180^°^ difference of the angles of the median peak total eye velocities of the excitatory and inhibitory phases, compared to totalVOR+. In other words, the eyes moved to the opposite direction than in totalVOR+. In BV2-PAN and BV3-LAN, inversion of the gyroscopes resulted in an inversion of the vertical peak total eye velocities, but not of the horizontal peak total eye velocities. For example in BV2-PAN: when the head was moving to the right in totalVOR+, the eyes moved downwards and to the left. When the head was moving to the right in totalVOR−, the eyes now went upwards, but remained moving to the left. To summarize, the resulting angle of the eye responses in the combined conditions was a non-linear mix of the responses resulting from residual “natural” input and “artificial” VI-input across subjects.

**Table 3:** Median angles (°) and confidence intervals in the excitatory and inhibitory phases of stimulation, of each electrode in each condition.

	Excitation				Inhibition			
	VOR	eVOR	totalVOR+	totalVOR−	VOR	eVOR	totalVOR+	totalVOR−
BV1-LAN	25.6 (12.5–39.2)	358.4 (4.5–345.9)	352.9 (348.8–355.3)	178.6 (173.7–188.4)	165.5 (147.7–173.6)	180.4 (163.6–188.6)	165.1 (157.6–173.5)	354.8 (351.4–359.7)
BV1-SAN	7.4 (18.1–353.8)	10.5 (0.0–15.1)	0.3 (5.6–357.6)	190.3 (187.3–193.5)	171.4 (167.4–185.8)	190.2 (180.6–195.7)	185.2 (182.0–191.6)	0.4 (5.4–358.1)
BV2-PAN	193.0 (190.0–200.0)	248.3 (243.4–254.9)	241.1 (229.9–244.3)	126.2 (111.3–131.8)	12.6 (6.2–15.5)	76.3 (75.1–78.7)	53.8 (45.6–65.4)	278.5 (269.0–283.6)
BV3-LAN	358.2 (3.9–349.6)	55.8 (52.5–59.0)	28.1 (25.8–37.0)	331.5 (313.6–352.0)	184.3 (176.3–191.5)	244.4 (227.5–255.4)	191.7 (188.0–199.7)	146.2 (122.6–152.8)
BV4-SAN	13.6 (182.9–188.1)	64.3 (88.0–90.8)	48.7 (99.0–101.7)	9.9 (268.1–275.9)	357.9 (2.5–354.8)	275.3 (272.2–277.2)	287.2 (285.5–290.2)	88.4 (86.5–89.7)

*VOR, vestibulo-ocular-reflex; eVOR, electrically evoked vestibulo-ocular-reflex*.

#### Asymmetry

**Figure 5:**
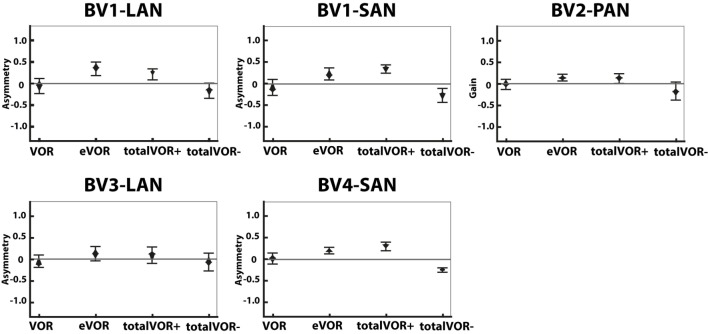
Asymmetry of the eye movement responses plotted for each electrode in each condition [vestibulo-ocular-reflex (VOR), electrically evoked vestibulo-ocular-reflex (eVOR), totalVOR+, and totalVOR−]. The widest part of each diamond represents the median, upper and lower parts of the diamond the 95% confidence intervals, and the bars the interquartile ranges. The amount of analyzed excitatory and inhibitory peak total eye velocities is the same for each electrode and condition as in Figure 4.

Figure 5 presents the asymmetry of the eye movement responses plotted for each electrode in each condition. A significantly higher asymmetry in eye movement response was found in eVOR compared to VOR in all electrodes (*p* ≤ 0.0002). When comparing VOR with conditions that involved “artificial” VI-input combined with residual “natural” input (totalVOR+ and totalVOR−), a significant asymmetry was found in totalVOR+ in almost all electrodes (*p* ≤ 0.001) except BV3-LAN, and in totalVOR− in BV2-PAN and BV4-SAN (*p* ≤ 0.0013). Asymmetry of responses of “artificial” VI-input only (eVOR) did often not significantly differ from totalVOR+ (only BV4-SAN, *p* = 0.0003), but always from totalVOR− (*p* ≤ 0.0011). In the combined conditions, the median asymmetry always inverted from a positive value in totalVOR+, to a negative one in totalVOR−. This asymmetry between totalVOR+ and totalVOR− significantly differed in almost all electrodes (*p* < 0.0001) except BV3-LAN. To summarize, “artificial” VI-input often introduced a significant asymmetry to the resulting eye responses across subjects.

#### Phase

**Figure 6:**
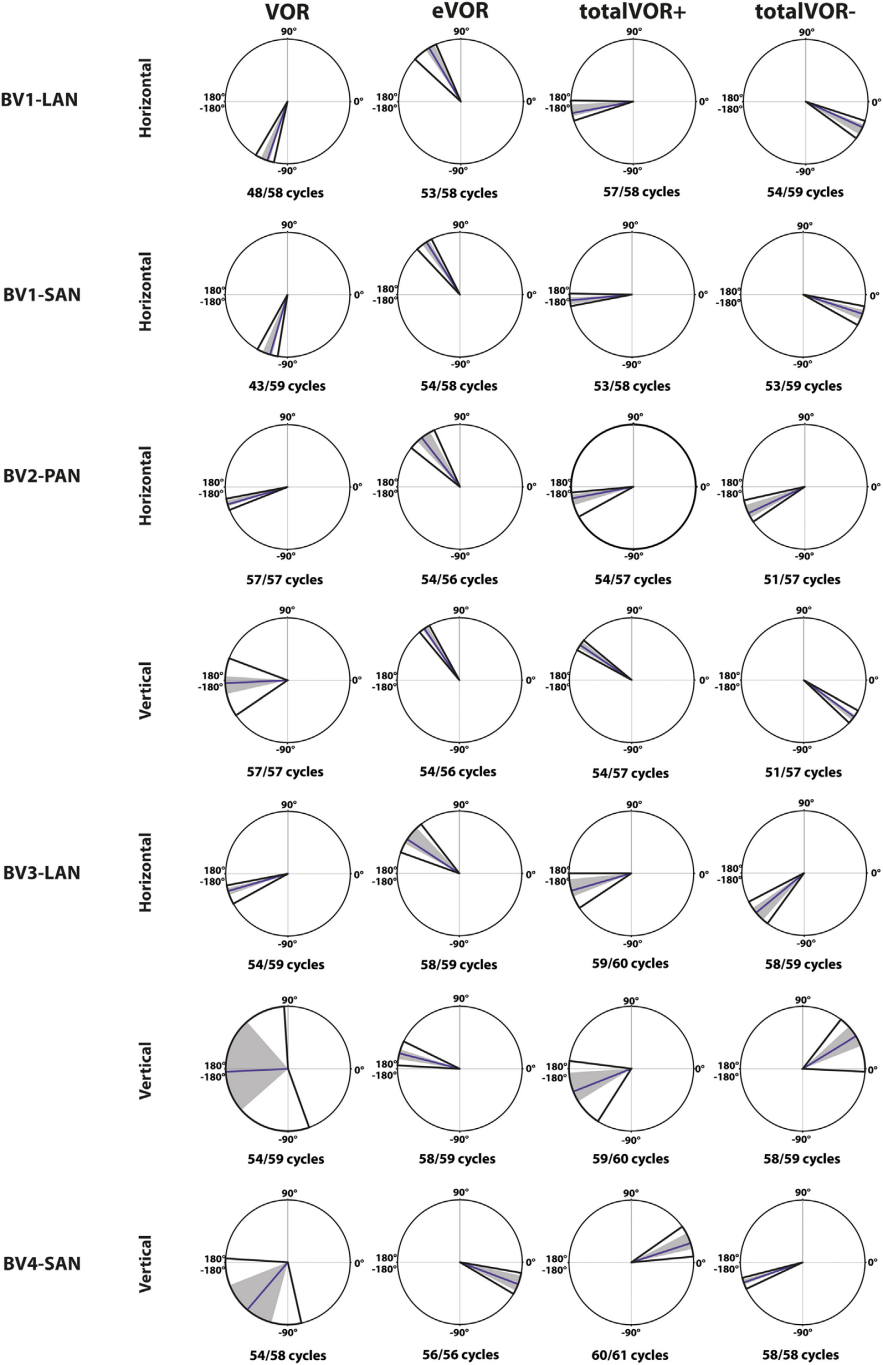
Phases of the eye movement responses plotted for each electrode in each condition [vestibulo-ocular-reflex (VOR), electrically evoked vestibulo-ocular-reflex (eVOR), totalVOR+, and totalVOR−]. Zero corresponds with “in phase,” ±180° with “counter phase.” Positive values correspond with a phase lead, negative values with a phase lag. The middle bar represents the median, the gray area the 95% confidence interval and the outer bars the interquartile ranges. Depending on the case, horizontal, vertical, or both phases are presented. The amount of cycles measured is given, as well as the amount of cycles available for analysis after data cleaning.

Figure 6 illustrates the phases of the eye movement responses plotted for each electrode in each condition. Regarding horizontal phases (horizontal phase of BV4-SAN not presented in this figure), all electrodes showed a phase lag in VOR and a “hypothetical” phase lead in eVOR (since the eye movement response was compared with a hypothetical horizontal head movement). They significantly differed from each other in all electrodes (*p* < 0.0001). In the predominantly horizontally aligned electrodes BV1-LAN and BV1-SAN, the median phase of the horizontal eye movement response in totalVOR+ was significantly more in counter phase (showing a phase difference closer to 180°) than the eye movement response in VOR and eVOR (*p* < 0.00001). No significant difference between VOR and totalVOR+ was found in the other electrodes with more vertical components in the response. Responses of “artificial” VI-input only (eVOR) always significantly differed from totalVOR+ regarding horizontal phases. Inversion of the gyroscopes in totalVOR− induced some clear phase shifts. In the electrodes with a predominantly horizontal response (BV1-LAN and BV 1-SAN), the horizontal eye movement response was almost in counter phase in totalVOR+, and almost in phase in totalVOR−. In other words, when the head was moving to the left in totalVOR+, the eyes were moving to the opposite direction, but when the head was moving to the left in totalVOR−, the eyes were moving in the same direction. In BV4-SAN (mainly vertical response), vertical phases were nearly in phase in totalVOR+, and nearly in counter phase in totalVOR−. In the electrodes BV2-PAN and BV3-LAN, horizontal phases did not differ significantly between VOR and totalVOR−. However, the vertical eye movement response changed from near counter phase (totalVOR+), to near in phase (totalVOR−). To summarize, the electrodes with a predominantly horizontal response were able to significantly decrease the phase lag of the residual “natural” function. Inverting the gyroscopes could introduce an almost 180° horizontal or vertical phase shift, depending on the electrode.

## Discussion

The main outcome of this study is the demonstration of significant interactions between the VI-generated VOR and residual “natural” function during the acute stimulation of vestibular nerve branches. It was shown that horizontal residual “natural” input was not sufficient to fully counteract the misalignment of the “artificial” VI-input. However, “artificial” VI-input was able to significantly influence the response to residual “natural” input.

**Figure 7:**
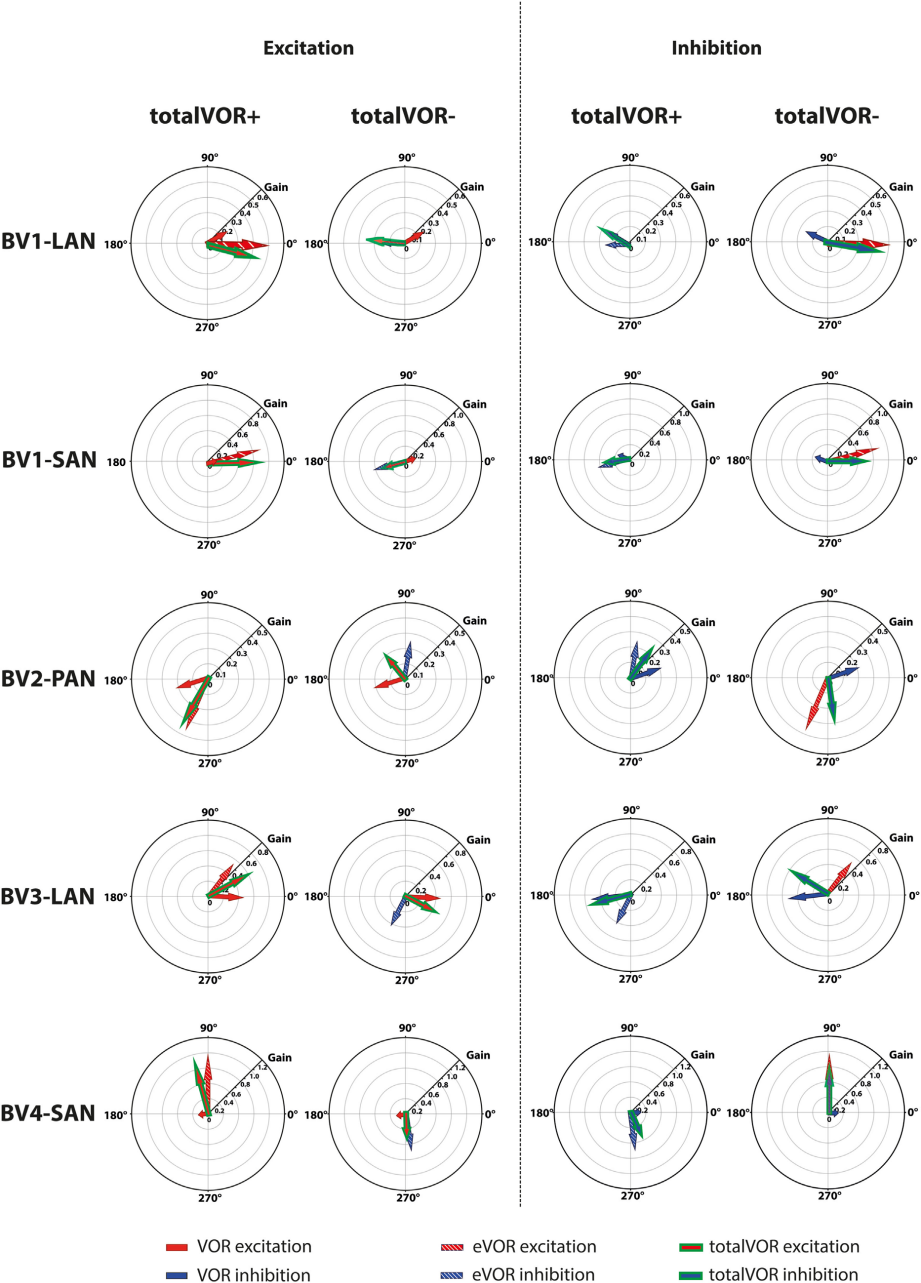
Schematic representation of the interaction between residual “natural” input and “artificial” vestibular implant input in the excitatory and inhibitory phases of the totalVOR+ and totalVOR− conditions of all electrodes. Arrows show the median vector of peak total eye velocities obtained during excitatory and inhibitory phases of the experimental trials. Excitatory vectors contain a red color; inhibitory vectors contain a blue color. Vestibulo-ocular-reflex (VOR) is represented by a plain arrow, electrically evoked vestibulo-ocular-reflex (eVOR) by a striped arrow, and totalVOR by a green edged arrow. In the excitatory phases of totalVOR+, the excitatory phases of VOR and eVOR were combined. In the excitatory phases of totalVOR−, the excitatory phases of VOR were combined with the inhibitory phases of eVOR, since the gyroscopes were inversed during the totalVOR− condition. In the inhibitory phases of totalVOR+, the inhibitory phases of VOR and eVOR were combined. In the inhibitory phases of totalVOR−, the inhibitory phases of VOR were combined with the excitatory phases of eVOR.

The interaction between “artificial” VI-input and residual “natural” input has not been extensively studied before in humans, although all BV patients can still have some residual “natural” input that can significantly contribute to the response. This study was designed as an initial exploratory test to investigate the possible interaction, by inducing conflicts between vectors of eye movements. It was illustrated that when both inputs combined (measured in VOR and eVOR conditions), an interaction occurred in which some of the characteristics of the resulting eye movement responses (totalVOR+ and totalVOR− conditions) significantly differed from one or both inputs. This interaction was not linear: the vectors obtained in the totalVOR conditions did not seem to be a clear linear summation of the vectors obtained in the VOR and eVOR conditions, as illustrated in Figure 7. It could be hypothesized that when vectors containing different components are combined, the “strongest” components might be represented more in the resulting totalVOR, than the “weaker” components. This could clearly be observed in the totalVOR− conditions of the “artificial” VI-inputs with an almost equal magnitude with respect to their corresponding residual “natural” inputs (BV2-PAN and BV3-LAN). In these cases, the resulting vectors of eye movements in totalVOR− showed angles of which the horizontal component was dominated by the input with the strongest median horizontal peak eye velocities, and the vertical component was dominated by the input with the strongest median vertical peak eye velocities (Figure 7). This was also found in the “artificial” VI-inputs with a much higher magnitude than their corresponding residual “natural” inputs (BV1-LAN, BV1-SAN, BV4-SAN): after combining both inputs, the resulting totalVOR conditions were often dominated by components of the “artificial” VI-input. Especially in the totalVOR− conditions of these electrodes, in which clear phase shifts were observed, it was shown that “artificial” VI-input was able to “counteract” residual “natural” input (Figure 7).

Some electrodes showed clear misalignment (e.g., BV1-SAN with a predominantly horizontal response). Although highly interesting, the background for this was previously extensively described and not within the scope of this article (2, 15, 17). However, this misalignment (next to purposely selecting electrodes with a predominantly vertical response and inverting the gyroscopes) facilitated an interaction between horizontal residual “natural” input and predominantly vertical and/or inversed “artificial” VI-input. Within this interaction, the brain was not able to fully suppress conflicting vestibular information in the acute phase of stimulation (32). In other words: horizontal residual “natural” input was not sufficient to fully counteract the misalignment of the “artificial” VI-input. In these cases, the central vestibular system did not thoroughly distinguish between input that was congruent with the axis of whole-body rotation (the residual “natural” input) or with input that was sometimes conflicting with the axis of whole-body rotation (the “artificial” VI-input). In animals, “cross-axis adaptation” has been described, in which vertical eye movement responses gradually shifted toward alignment with the axis of horizontal head rotation after long periods of chronic use. This indicates that the central nervous system rapidly adapts to VI-input (33). Cross-axis adaptation takes several days to occur in animals (2, 27). In humans, it has not been reported for stimulation periods longer than these 60-cycle trials, in which cross-axis adaptation did not occur in the electrodes with predominantly vertical eye responses. This should be evaluated in future studies involving longer periods of chronic stimulation. However, the interaction between residual “natural” input and “artificial” VI-input could possibly enhance faster adaptation than previously described in animals (19, 27, 33, 34). In case the human brain is not able to correct for the misalignment after adaptation during chronic use, more complex stimulation paradigms might be necessary.

These findings do imply that the response to residual “natural” input can be influenced by “artificial” VI-input. This might pave the way for using the VI as a “vestibular pacemaker.” Future studies could address this subject by using the VI to reduce vestibular asymmetry as occurring during disabling attacks of vertigo (10). However, whether this interaction is beneficial or counterproductive in these situations has not been determined yet.

In most electrodes, “artificial” VI-input significantly increased gain in totalVOR+ (the condition in which the VI should work in daily life). The totalVOR response did not seem to result from a simple, linear interaction between VOR and eVOR responses (e.g., the horizontally aligned electrodes BV1-LAN and BV1-SAN; Figure 4). Next to this, gain did not often reach the value of 1. This was due to a lower response to electrical stimulation, as well as the natural frequency-dependency of the vestibular system. After all, gain in healthy individuals does not necessarily have to reach the value of 1 at 1 Hz modulation (17). Furthermore, in the electrodes generating a predominantly horizontal eye movement response, phase could significantly be restored. This is an important finding since BV patients often show phase abnormalities in their VOR response (1). The improvement of gain and phase of the eye movement response in totalVOR+ (the condition in which the VI should work in daily life) is encouraging for the future rehabilitation prospects of the VI. A previous study of our group also supported this by showing a significantly increased performance during a real-life task (walking), resulting from VI-stimulation (35).

An additional important finding of this study was the observation of non-linearities induced by VI-stimulation. “Artificial” VI-input (present in eVOR, totalVOR+ and totalVOR−) often resulted in asymmetrical shapes of eye movements, not fully replicating the shape of the sinusoidal stimulation signal. This implies that traditional methods of signal analysis like fitting with a sinus or application of Fast Fourier transform (14, 20) might be insufficient for evaluation of eye movements obtained by the VI. Several facts could contribute to this asymmetry. Firstly, a linear transfer function was chosen, from the lower threshold, to baseline, to the upper threshold. However, neural responses to electrical stimulation rarely follow a linear relationship (36). Secondly, the VI unilaterally stimulates the ampullary nerves in a non-physiological way: it involves a relatively non-selective electrical stimulus that bypasses all biophysical properties of the peripheral end-organ and selected pulse rates were supra-physiological (37). Thirdly, another contributing factor could be the paradigm of determining the dynamic ranges: the lowest threshold for stimulation was determined as the first (lowest) level of electrical current where the first vestibular symptom was observed or reported (15). However, in recent experiments it was observed that perception can have a lower threshold than the VOR (38). This implies that when baseline stimulation is set at half the dynamic range, it might not be halfway the dynamic range for eliciting a VOR. This could result in an asymmetrical eVOR response since the eVOR might reach saturation earlier during the inhibitory phase of stimulation than during the excitatory phase of stimulation.

### Other Considerations and Limitations

Patients in this study still had some residual “natural” input. They fitted the stringent inclusion criteria for implantation, including a gain of less than 0.25 on rotatory chair tests using the typical clinical frequency of 0.1 Hz (14). By increasing the stimulation frequency to 1 Hz, gain of the residual “natural” input increased (17). This facilitated a higher residual “natural” input, to interact with the “artificial” VI-input. It also explains why some patients (e.g., BV3) showed a higher VOR response than would initially be expected from the inclusion criteria.

Motivation for not using a bite bar was previously described (17). Unfortunately, head movement artifacts (vertical head movements or differences in velocity) were observed in some VOR, totalVOR+, and totalVOR− conditions. These unwanted head movements affected mainly angle. Therefore, no statistical analysis was performed regarding angle of the obtained eye movements.

Only a 1 Hz sinusoidal stimulus was chosen for this study. It was previously shown that the eVOR has an acceptable gain at 1 Hz compared to lower frequencies where alertness and arousal may influence the gain substantially more. Next to this, head fixation to the rotatory chair becomes more necessary at higher frequencies to avoid artifacts by head inertia. In contrary to lower frequencies, patients’ arousal is also less compromised during 60-cycle trials at 1 Hz. Besides, other parameters like angle and asymmetry show no significant frequency dependency and habituation does not play a key factor in the eVOR analysis when using this paradigm (17). However, this also implies that the findings of this study might be specific for this frequency (39, 40) and that they cannot directly be extrapolated to other frequencies.

## Conclusion

In the acute phase of VI-activation, residual “natural” input and “artificial” VI-input interact to generate eye movement responses in a non-linear fashion. The observed interaction implies that different stimulation paradigms and more complex signal processing strategies (e.g., non-linear transfer functions) will be required unless the brain is able to optimally combine both sources of information after adaptation during chronic use. Next to this, these findings could pave the way for exploring the use of the VI as a “vestibular pacemaker.”

## Ethics Statement

This study was in accordance with the Declaration of Helsinki (amended version 2013). Approval was obtained from the ethical committees of Maastricht University Medical Center (NL36777.068.11/METC 11-2-031) and Geneva University Hospitals (NAC 11-080). All participants provided written informed consent prior to the study.

## Author Contributions

All authors contributed extensively to the work presented in this article. RB wrote the manuscript. RB, NG, MR, SC, KN, and AP-F performed the experiments. RB, HK, DS, and MH developed and performed the analysis. FL created and edited the figures of the manuscript. NG, MR, SC, KN, J-PG, FL, RS, HK, MH, and AP-F reviewed and edited the manuscript the manuscript.

## Conflict of Interest Statement

Med-El has provided the VCI-devices, funding for research (not presented here), and funding for travel. H. Kingma and R.v.d.Berg are supported by the Russian Science Foundation (project No. 17-15-01249).
